# Supplementary material for: Near-Infrared Photothermally Enhanced Photo-Oxygenation for Inhibition of Amyloid-β Aggregation Based on RVG-Conjugated Porphyrinic Metal–Organic Framework and Indocyanine Green Nanoplatform
Source: Int J Mol Sci. 2022 Sep 17;23(18):10885. doi: 10.3390/ijms231810885 (PMC9505608; doi:10.3390/ijms231810885)
Supplement: Supplementary file 1 [file ijms-23-10885-s001.zip › ijms-1881132-Supplementary.pdf]

Supporting Information

Near-Infrared Photothermally-Enhanced Photooxygenation Inhibition of Alzheimer's Amyloid-β Aggregation Based on Brain-Targeting Peptide RVG conjugated Two-dimensional Porphyrinic Metal-Organic Framework@Indocyanine Green (PCN-222@ICG@RVG) Nanoplatform

Jiuhai Wang, Yutian Gu, Xu Liu, Yadi Fan, Yu Zhang, Changqing Yi, Changming Cheng, Mo Yang[*]

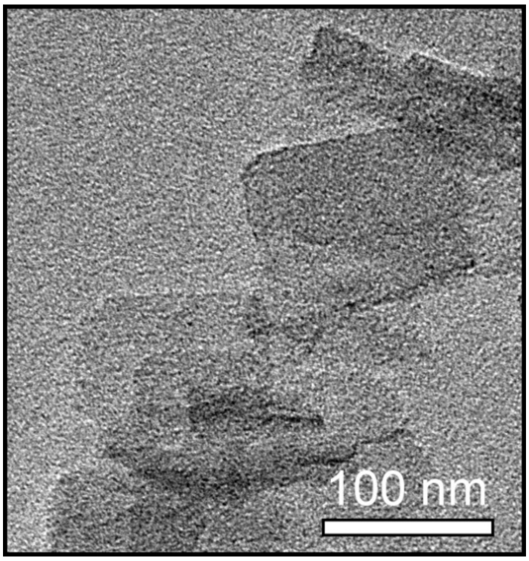

Figure S1. TEM image of PCN-222 nanosheet.

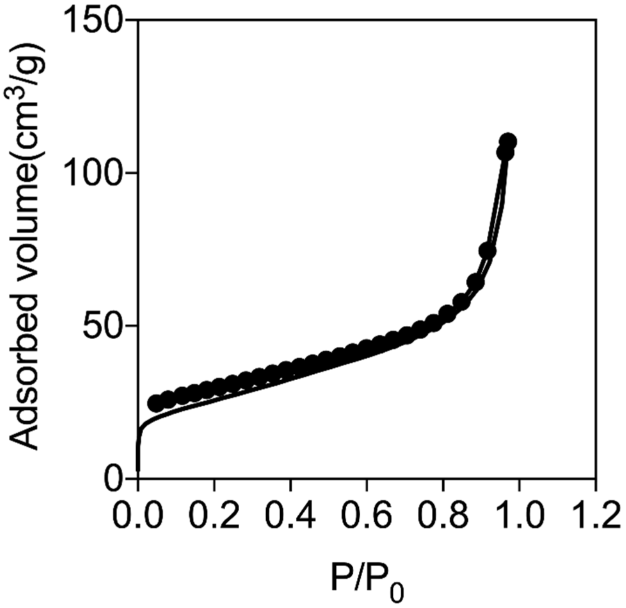

Figure S2. N_2 adsorption/desorption spectrum of PCN-222@ICG.

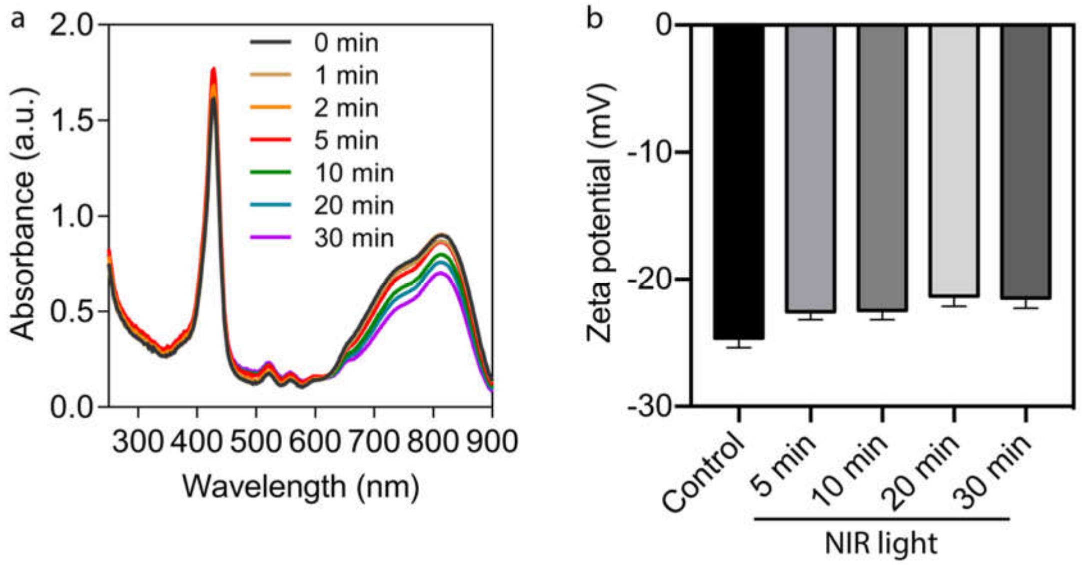

Figure S3. Photostability of PCN-222@ICG nanosheet. (a) PCN-222@ICG was irradiated by 808 nm NIR laser (0.6 W cm^{-2}) for 30 min and then measured by UV-vis spectrometer. (b) PCN-222@ICG was irradiated by 808 nm NIR laser (0.6 W cm^{-2}) for 5, 10 20, and 30 min and zeta potential was recorded.

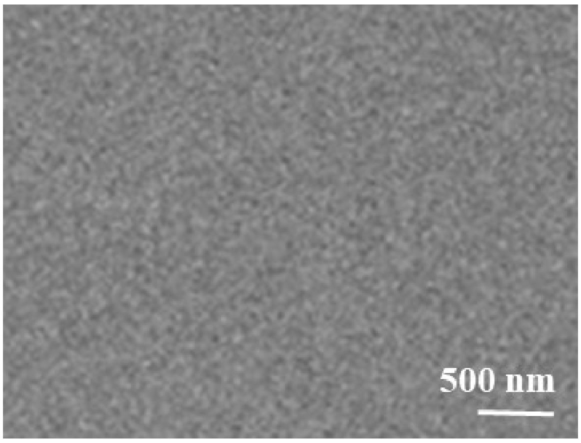

Figure S4. TEM image of $A\beta_{42}$ monomers at 0h.

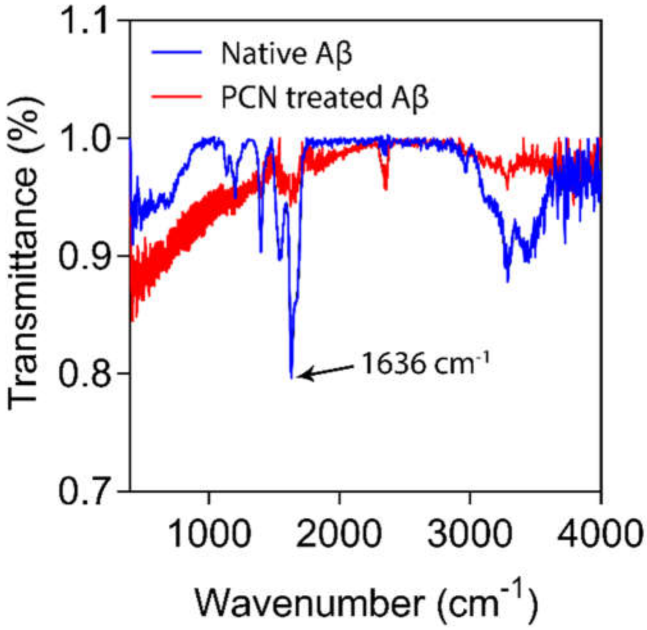

Figure S5. FT-IR spectrum of native $A\beta_{42}$ and $A\beta_{42}$ treated by NIR light in the presence of PCN-222@ICG.

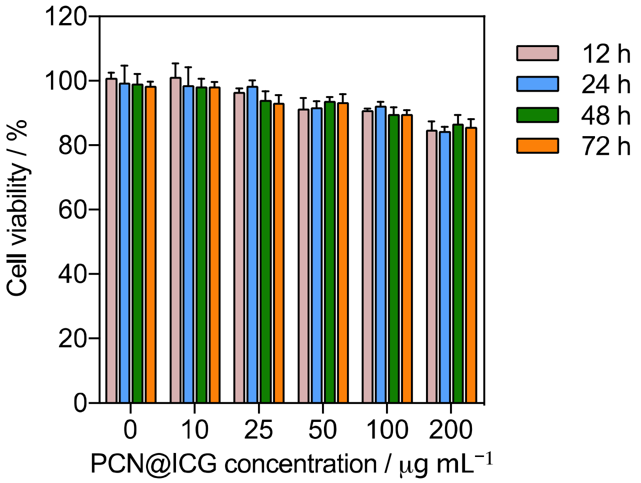

Figure S6. Cytotoxicity of PCN@ICG. PC 12 cells were incubated with PCN@ICG at various nanoparticle concentrations for 12 h, 24 h, 48 h, and 72 h, and the viability was determined by CCK-8 assay.

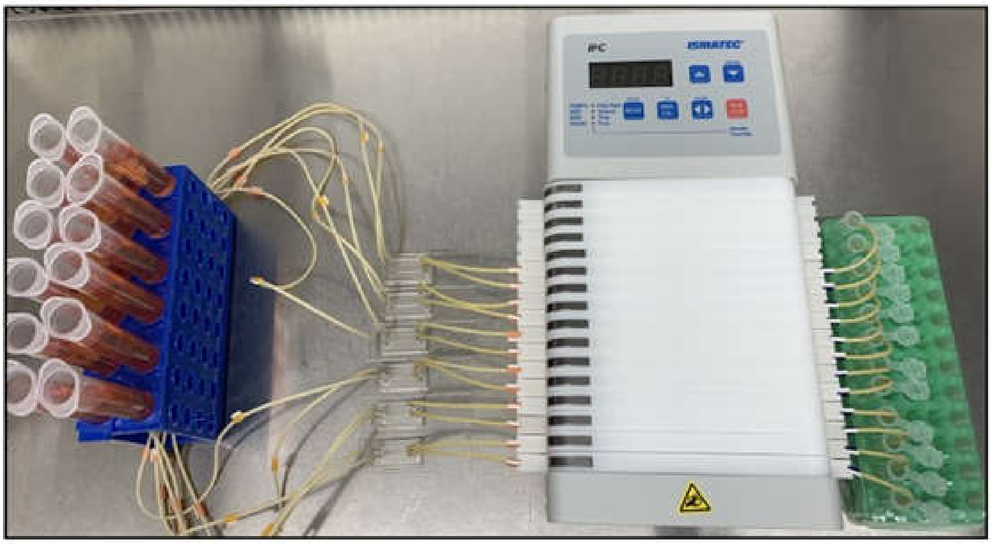

Figure S7. Dynamic culture system mimicking physiological environment provided by a peristaltic pump.

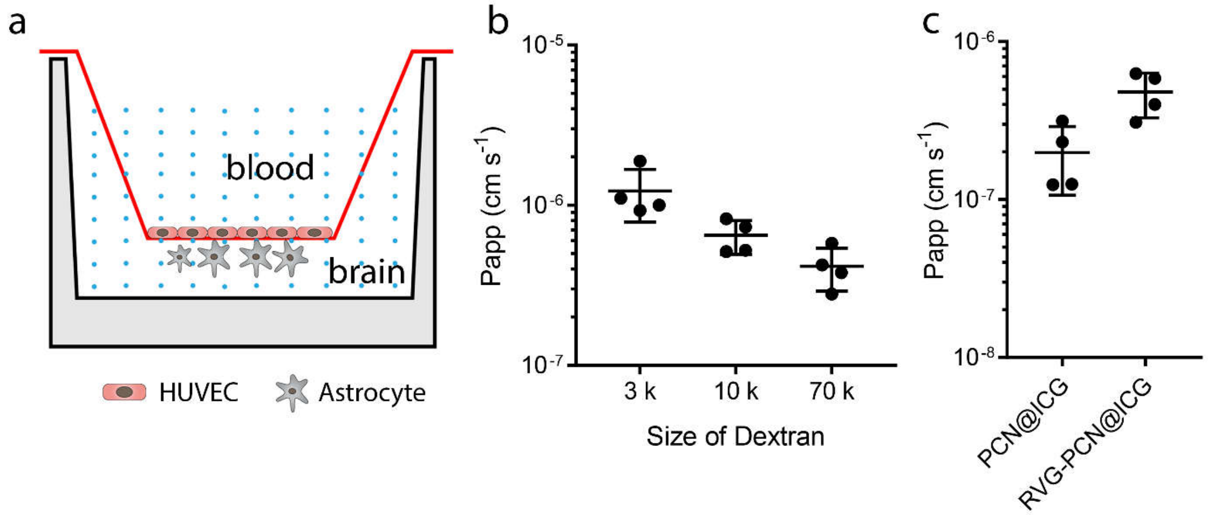

Figure S8. (a) *In vitro* BBB Transwell model with HUVEC and Astrocytes cultured on the upper and lower side of Transwell membrane, respectively. (b) 3 k, 10 k, 70 k dextran were dosed to blood side to monitor the tightness of BBB. (c) BBB translocation rate was studied by dosing PCN@ICG and RVG-PCN@ICG (0.025 mg mL^{-1}) nanoprobes into the blood side of the Transwell model for 12 h.
